# Noncontact and Nonintrusive Microwave-Microfluidic Flow Sensor for Energy and Biomedical Engineering

**DOI:** 10.1038/s41598-017-18621-2

**Published:** 2018-01-09

**Authors:** Mohammad Hossein Zarifi, Hamid Sadabadi, S. Hossein Hejazi, Mojgan Daneshmand, Amir Sanati-Nezhad

**Affiliations:** 1 BioMEMS and Bioinspired Microfluidic Laboratory, Department of Mechanical and Manufacturing Engineering, University of Calgary, Calgary, Alberta T2N 2N1 Canada; 2 Department of Electrical and Computer Engineering, University of Alberta, Edmonton, AB T6G 1H9 Canada; 3 Department of Electrical Engineering, University of British Columbia, Kelowna, BC V1V 1V7 Canada; 4 Subsurface Fluidics and EOR Laboratory, Chemical and Petroleum Engineering, University of Calgary, Calgary, AB T2N 1N4 Canada; 5 Center for BioEngineering Research and Education, University of Calgary, Calgary, AB T2N 1N4 Canada

## Abstract

A novel flow sensor is presented to measure the flow rate within microchannels in a real-time, noncontact and nonintrusive manner. The microfluidic device is made of a fluidic microchannel sealed with a thin polymer layer interfacing the fluidics and microwave electronics. Deformation of the thin circular membrane alters the permittivity and conductivity over the sensitive zone of the microwave resonator device and enables high-resolution detection of flow rate in microfluidic channels using non-contact microwave as a standalone system. The flow sensor has the linear response in the range of 0–150 µl/min for the optimal sensor performance. The highest sensitivity is detected to be 0.5 µl/min for the membrane with the diameter of 3 mm and the thickness of 100 µm. The sensor is reproducible with the error of 0.1% for the flow rate of 10 µl/min. Furthermore, the sensor functioned very stable for 20 hrs performance within the cell culture incubator in 37 °C and 5% CO_2_ environment for detecting the flow rate of the culture medium. This sensor does not need any contact with the liquid and is highly compatible with several applications in energy and biomedical engineering, and particularly for microfluidic-based lab-on-chips, micro-bioreactors and organ-on-chips platforms.

## Introduction

Microfluidic techniques have been extensively used for efficient manipulation of fluid flow in microscale for biomedical research and analytical chemistry. The control of flow in microfluidic networks is crucial for cell sorting, cell collection, flow mixing, cell adhesion and culture, droplet manipulation and flow driving^1^. Moreover, the flow rate needs to be accurately quantified to determine the concentration of cells^2^, and production of hollow microspheres^3^, droplets^4^, liposomes^5^, and chitosan microfibers^6^. A slight change in flow rate may lead to a size variation in the products. To precisely handle fluids at the microscale, the real-time detection of flow rate in microfluidic environment is essential and urgently needed though challenging.

Organ-on-a-chip (OOC) technology, in particular, aims to build biomimetic *in vitro* physiological micro-organs to compliment animal models in biological systems and benefit the pharmaceutical industry for drug discovery^7,8^. Many groups including ours have developed OOC platforms made of microbioreactors and integrated sensors for long-term and real-time monitoring the microenvironment, screening the status of miniaturized organs, and characterizing the response of micro-tissues to drugs^9–13^. The real-time measurement of heat transfer^14^, differential pressure^15^, pH and oxygen^11^ and biomarkers^10^ are central to biomimetic performance of OOC systems. Miniaturized biosensors provide favorable features like low-cost reagents consumption, decreased processing time, reduced sample volume, laminar flow to cells, parallel detection for multiple samples as well as portability^12,13,16^. However, the OOC systems still need on-chip integrated flow sensors compatible with their fabrication processes and functions^17^. The OOC platforms require the design of appropriate systems with controlled fluidic conditions for generating *in vivo*-like flow patterns as well as fluidic circulation systems to interconnect multiple organs in a physiologically relevant scheme. The flow rate in microfluidic bioreactors is usually controlled by commercially available syringe/peristaltic pumps. However, there is an evident delay from the pumping site to the target area within the chip, especially with longer channels and tubes and under low flow rates. The continuous measurement of flow rate can also be instrumental for biosensors integrated to microfluidic bioreactors to provide in-line measurement of flow fluctuation in microfluidic biosensors and achieve high-resolution detection of biophysical parameters and biochemical analytes. The flow sensing would be more critical for multi-organ platforms where the cross-talk of different bioreactors and biosensors changes the fluid flow among components and affects the accuracy of measurements in microfluidic devices^10^. The other challenge is an engineering obstacle for maintaining a precise fluid volume over each organ model for a certain period while operating at relatively high fluid exchange rates. Other issues such as the evaporation of culture medium, slight variations in pump stroke volume, leakage, bubble generation due to the diffusivity through the polymer, and the very low volume of culture medium in the microfluidic network highlights the necessity of long-term and real-time monitoring the flow rate over the course of cell culturing and drug testing. Moreover, perfusion systems such as autonomous capillary action, negative pressure system, gravity flow and micro-pipetting are preferred opposed to positive hydrodynamic pumping systems because of the small size of channels and the detrimental effect of shear stress on cell viability. However, the gravity flow system has constraints in proper controlling the dynamic flow which necessitates real-time and local measurement of flow rate in microfluidic bioreactors^18,19^. On-chip flow sensors that are non-invasive, easy to fabricate and integrable would significantly enhance the functionality of microfluidic bioreactors and facilitate the development of biomimetic models mimicking the *in vivo* microenvironment.

The ideal flow sensor for OOCs would measure the flow rate locally in a long-term, continuous and fully automated manner, with minimal side effects on flow patterns, cells cultured within bioreactors, and functions of other integrated sensors. Off-chip flow sensors can be integrated to microchips but they cannot measure localized flow alterations and may interrupt the normal flow patterns in microsystems. Also their integration to microfluidics for measuring the flow rate at multiple points of the fluidic system is challenging^20–22^. The micro-particle image velocimetry (micro-PIV) is not suitable for long-term flow rate detection and requires a continuous flow of particles through the circuit, which is not applicable for microfluidic bioreactors. Also the long-term monitoring the flow rate in particle tracing-based flow measurement systems requires an access to a high-resolution microscope with motorized stages and autofocus function to sequentially record the movement of particles or liquid – gas interfaces with no Z drift^9^.

The primary microfluidic-integrable flow sensors are micro electromechanical systems (MEMS)-based sensors with high-resolution sensing performance. However, the MEMS flow sensors have the constraint in complexity of fabrication processes and the drawback of contact-based measurement^23,24^. Other types of integrable flow sensors are thermal anemometers^25^, optical devices^26^, and electrical admittance sensors^27^. These flow sensors, however, have limitations such as the complexity of integration and multi-steps stacking, fiber tapering as well as time-consuming and costly fabrication. Also for achieving high sensitivity, their performance is affected by flow fluctuations induced by thermal energy and bubble generation at high power sources^28^.

The *In-situ* deformable elements such as deformable springs, beams, cantilevers and membranes with a highly controlled mechanical behavior provide a direct indication of flow rate^29^. Some of these cantilever-based flow sensors such as polydimethylsiloxane (PDMS) microcantilevers are easily integrable with lab-on-chip systems^24^ but they require integration of additional sensing components such as chrome-coated surface^30^, fluorescent particles^31^, micro-sized silver powder^32^, ionic liquid^33,34^, or measurement of deflection with optical photo-diode (PSD) or piezoresistive read-out, which make their integration to bioreactors very complex^35–38^. Also these deformable elements are sensitive only within a limited range of flow rates due to the limitation in their detection system like the reflection of laser beam on the detector^26^. The flow sensors made of pillars containing iron nanowires were developed from polymers with a high stability and silicon with a high sensitivity. While the detection system is microscope-free (magnetic-based), the fabrication process is not compatible with the multilayer, soft-lithography processes of OOCs^39^. Also the pillars embedded within the microchannels may interrupt the flow and block the channels during the flow of single cells. Moreover, nanocomposite materials may release into the culture medium and circulate through the system, interrupting the normal cells function. The large magnetic fields may also impact the cells cultured within microbioreactors at the vicinity of sensing components^9^.

Recently, microwave planar resonator devices have demonstrated promising results for sensing applications. They operate based on the interaction of electric fields with materials in the sensor’s near soundings. The dielectric properties of materials (permittivity and conductivity) affect the electric field and consequently electrical properties of the resonator such as the resonant amplitude, resonant frequency and quality factor^40–42^. The planar structure, simple fabrication process and robustness of microwave resonators make them attractive for a variety of different applications, such as liquid monitoring in oil-sand^43,44^, gas sensing for environmental monitoring^45^ and studying nanomaterials and nanostructures^43^. These microwave and impedance-based measurement systems have also measured the flow rate within channels and tubes but they have used the flow discontinuity in form of droplets or particles transported with respect to the support fluid. The utilization of such discontinuity is challenging for bioreactors and OOCs due to their harmful impact on cultured cells. All above, the existing on-chip integrated flow sensors are not suitable for miniaturized bioreactors and OOCs. There are yet challenges for the development of flow sensors compatible with complex microfluidic bioreactors and the long-term and real-time monitoring the flow rate with minimal limitations in system-level integration and full automation.

In the present work, a robust, portable and scalable flow sensor relying on microfluidic and microwave technologies is presented for the real-time, long-term, noncontact and nonintrusive detection of flow rate in microfluidic environment. The flow sensor detects the flow rate with the resolution of 1 µl/min, with the detection limit of 0.5 µl/min, within the detection range of 0.5–300 µl/min. The high performance of this sensor is sourced from the high sensitivity of the integrated thin circular membrane to the pressure change resulted from the fluid flow; the specific design of microwave platform; and the presence of the *thin* membrane interfacing the electrodes and the fluid. Compared to other lab-on-chip compatible flow sensors, this novel flow sensor has the advantages of (a) providing a noncontact mode for integration to measure a wide range of flow rates in a reasonably linear response, and (b) high sensitivity and non-intrusiveness performance that can be highly beneficial for OOCs. The flow sensor demonstrated its long-term performance for monitoring the flow rate of cell culture medium stably and with compatibility with the environment of cell culture incubators. The simulation results confirmed that the flow rate functions noninvasively within micro-bioreactors, making it a harmless flow sensor for OOC platforms. While we focused on the flow sensing specific for OOCs, the accurate and high-speed measurement of complex permittivity of fluidics such as ethanol and water is also demonstrated to show its broad applications for biomedical and energy sectors with the potential of in-line assembly to microfluidics of any kind.

## Results and Discussion

### Deflection of the membrane and measuring the fluid flow

**Figure 1:**
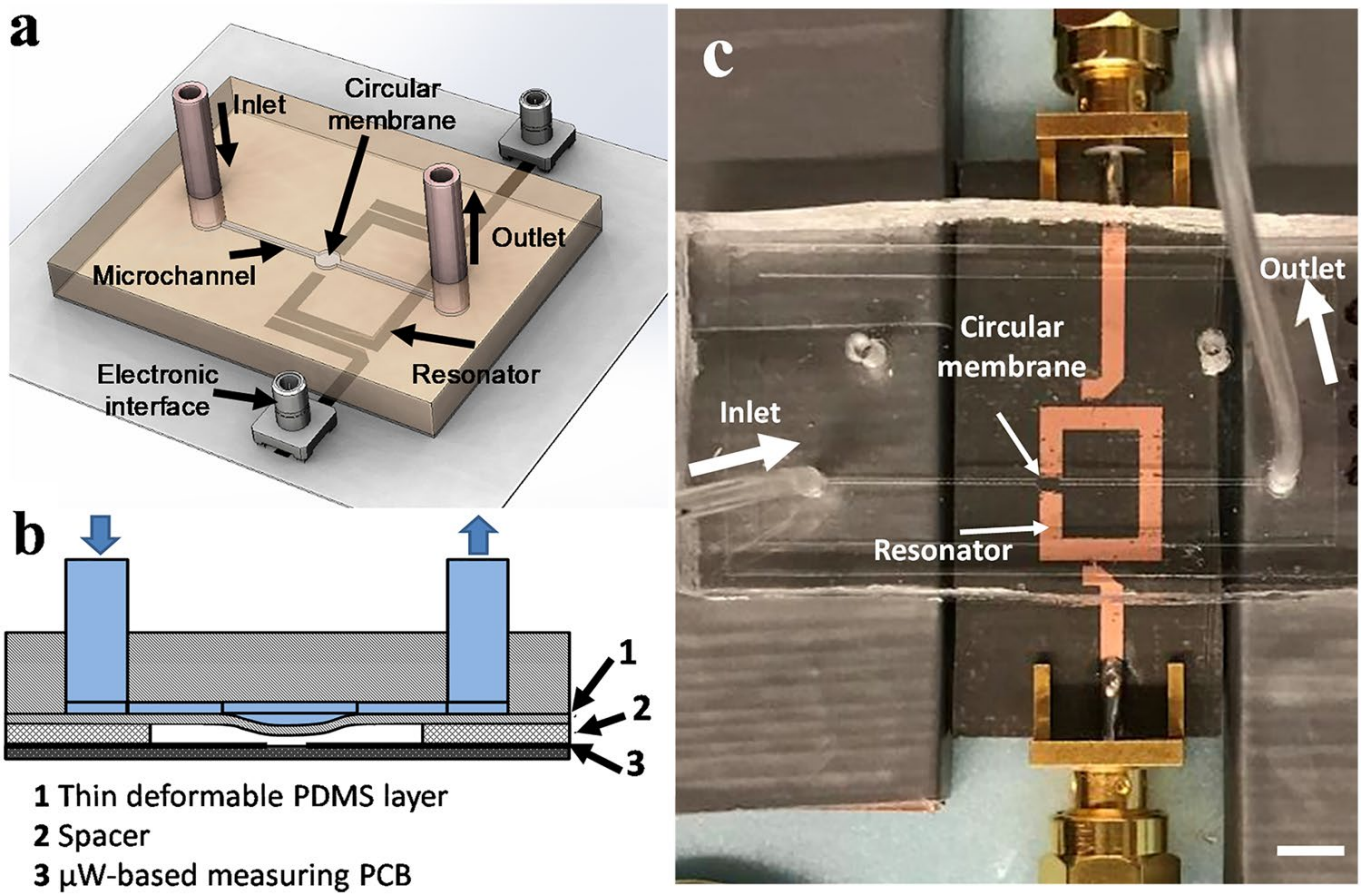
The microwave-microfluidic flow sensor. (**a)** Schematic design of the flow sensor integrating the microfluidic chip with the microwave resonator. (**b)** The flow through the microchannel deforms the thin polymer layer detectable by the change of permittivity of the environment measured by the resonator placed underneath. (**c)** The fabricated microwave-microfluidic resonator. Scale bar: 5 mm.

The flow of liquid through the microchannel passing over the thin circular membrane deforms the membrane and alters the effective permittivity of the medium above the sensor (Fig. 1).

**Figure 2:**
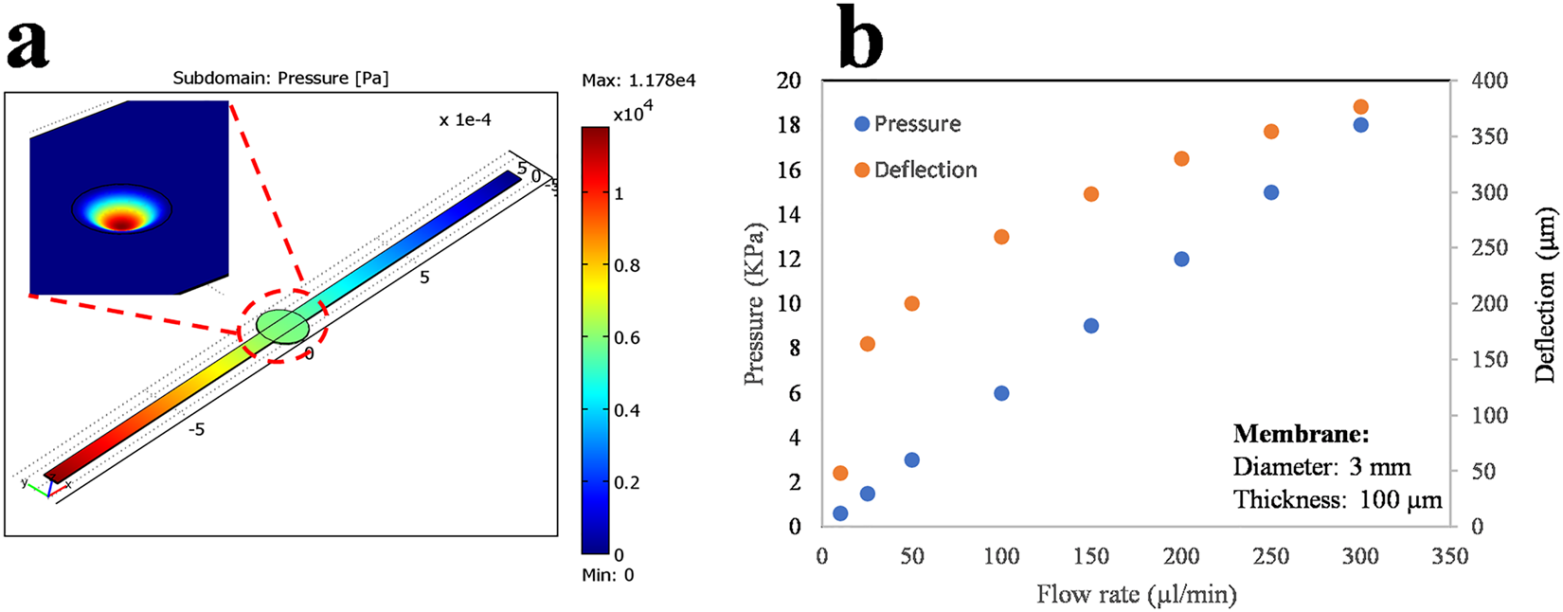
The numerical simulation of the membrane deformation. (**a)** The pressure distribution within the channel and the deformation of the large membrane (3 mm diameter) under the flow rate of 100 µl/min. The flow rate of 100 µl/min produced a flow pressure of 6 KPa over the membrane, resulted in a maximum deflection of 260 µm. (**b)** The numerical simulation of the membrane deformation (diameter: 3 mm, thickness: 100 µm) in response to different flow rates in the range of 10–300 µl/min.

The Reynolds number remains below 0.1 within the flow range of 1–300 µl/min tested in this work, therefore the laminar flow condition remains valid for the flow simulation purposes. Based on the simulation of Navier-Stokes and continuity equations as well as the force applied to the membrane under constant pressure, the deformation of the 3 mm diameter membrane with the thickness of 100 µm is presented in Fig. 2a. Given the small gap of 400 µm devised between the electrodes surface and the circular membrane in the non-deformed state, the deformed membrane bulges freely for the flow rate range of 0–300 µl/min, where the deflection remains below 400 µm (Fig. 2b). The deformation of the large membrane at the flow rate of 100 µl/min is shown in Fig. 2a. For the flow rates of above 330 µl/min with the minimum deflection of 400 µm at the center of the large membrane, the membrane is subjected to an upward force resulted from the interaction of the membrane and the electrode surface.

Against all channel-suspended cantilever-based flow sensors, this membrane flow sensor is embedded at bottom of the microchannel, where the flow rate right at the surface of the membrane is zero and therefore its deformation is independent from the parabolic nonlinear flow profile over the circular membrane^24^.

### The electromagnetic field of resonator and the flow rate

**Figure 3:**
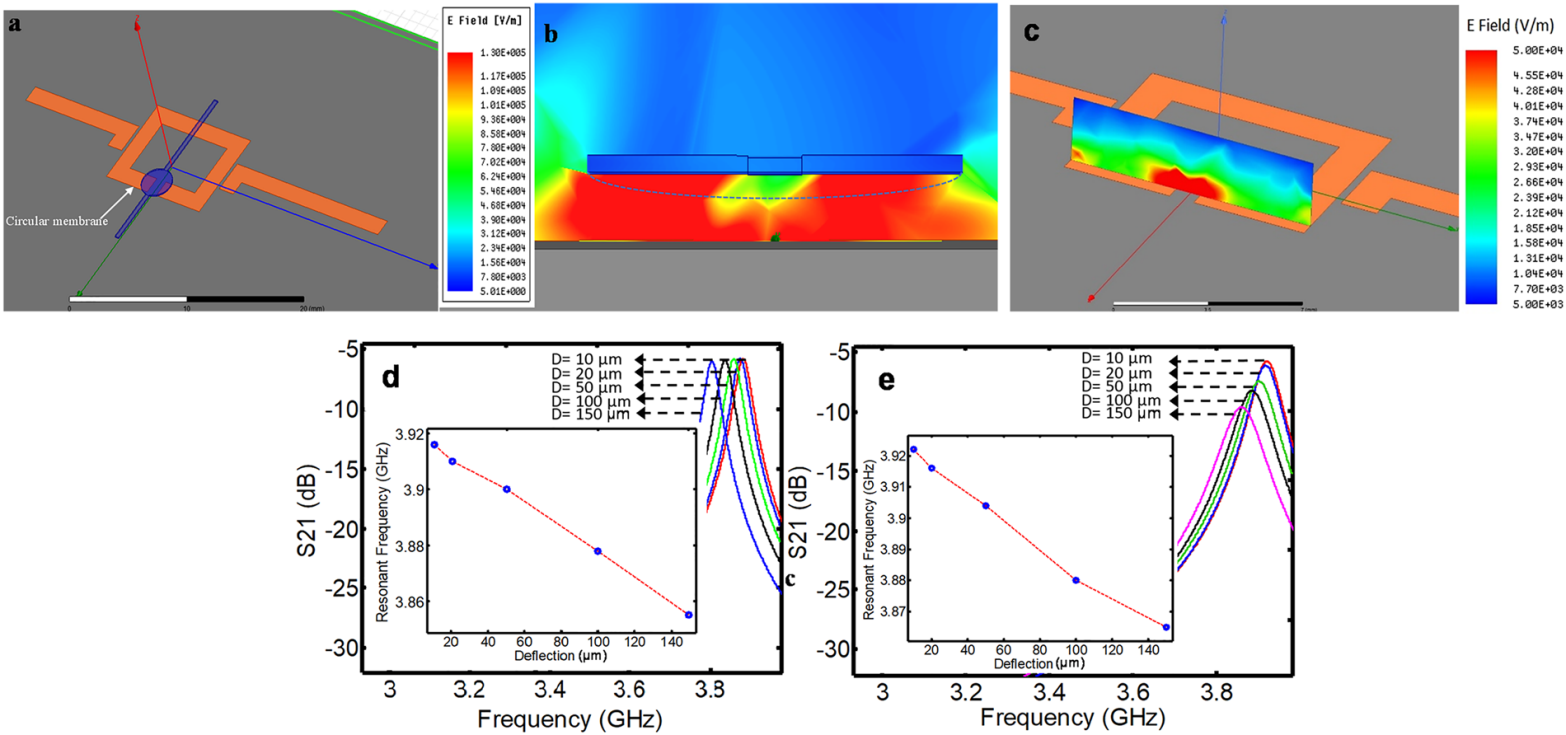
Changes in electric field of the resonator versus bulging of the circular thin membrane. (**a**) The implemented microfluidic-microwave sensor in HFSS. (**b**) The electric field distribution in front of the resonator in the sensitive region at the resonant frequency. (**c**) The HFSS simulation confirms that the electric field is 10 times smaller at 3 mm distance from the resonator sensor than its value on the resonator surface. Therefore, the flow sensor can measure the flow rate noninvasively within microchannels. (**d**) The resonant profile (S21) for different deflection of the membrane with water content (ε = 79, tanδ = 0.02). (**e**) The resonant profile (S21) for different deflection value of the membrane with ethanol content (ε = 16, tanδ = 0.02).

According to the field distribution in proximity of the resonator, any deflection (bulging) of the thin membrane changes the effective permittivity experienced by the resonator, and as a result, alters the effective capacitance of the resonator and the electrical parameters of the sensor such as resonant frequency and resonant amplitude. Since the microwave planar resonators are operational based on the dielectric properties of the medium over the sensitive region of the microwave sensor, the type of liquid inside the microfluidic channel impacts the frequency response of the resonator (Fig. 3a,b). HFSS electric field simulation is performed to demonstrate the field variation along the axis perpendicular to the surface of the resonator device. According to the results, the electric field is 10 times smaller at 3 mm distance from the resonator sensor than its value on the resonator surface (Fig. 3c). Therefore, the HFSS simulation confirms that the sensor can measure the flow rate noninvasively within micro-bioreactors, making it a harmless flow sensor for OOC platforms. A simulation is also performed for two different liquids of water as the base material and ethanol as an arbitrary liquid, where water (ε = 79, tanδ = 0.02) and ethanol (ε = 16, tanδ = 0.02) are introduced into the microchannel. Also HFSS simulation illustrates that as the bulging increases, the variation for both liquids shows decreasing linear behavior (Fig. 3d,e inset). As expected, as the bulging increases, the effective permittivity increases and therefore the resonance frequency reduces.

**Figure 4:**
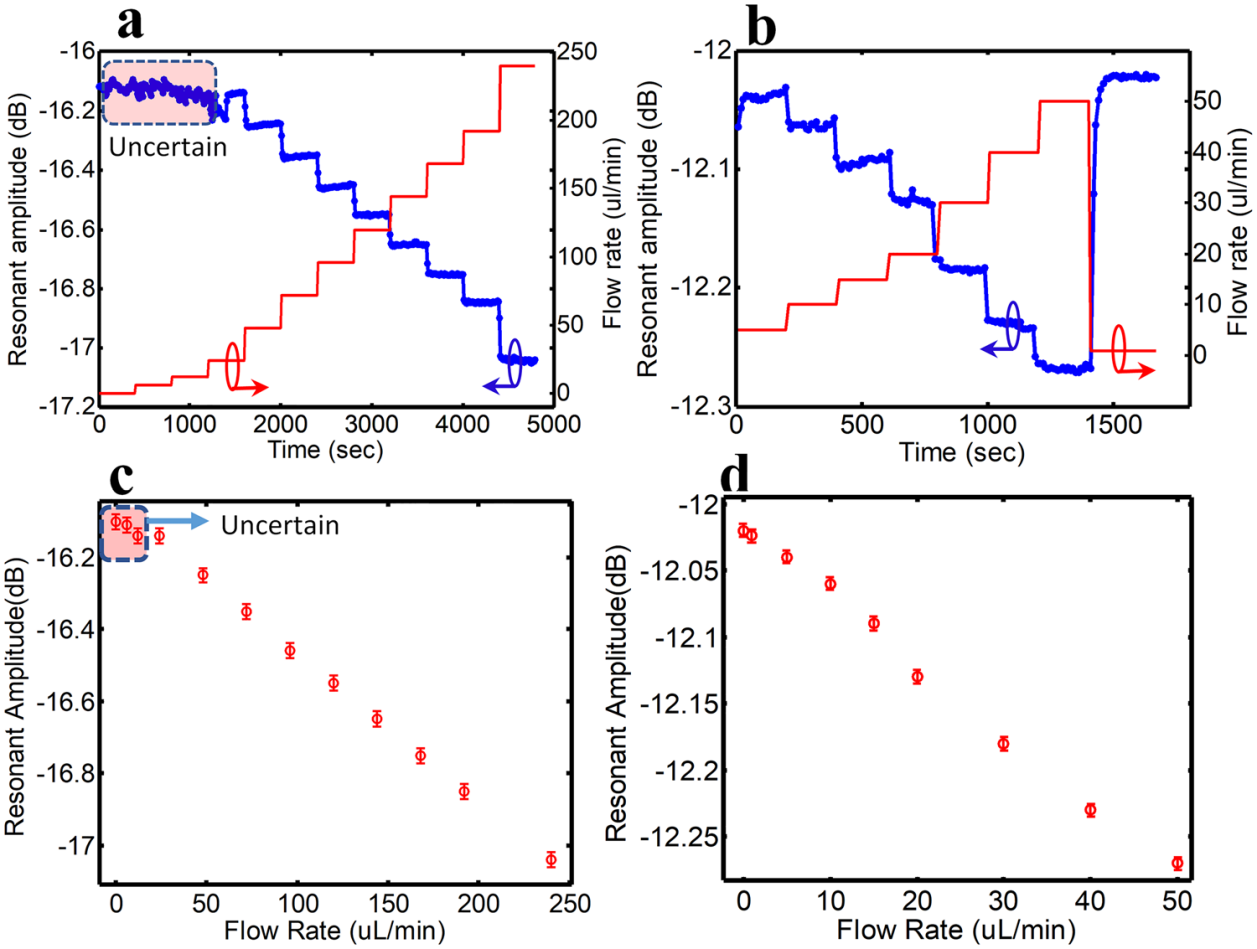
The response of the flow sensor to different flow rates. (**a)** The behavior of large membrane (3 mm diameter) integrated to the microwave resonator sensor in response to flow-rates versus time. The resonant amplitude is presented in blue and the flow rate is presented in red. (**b)** The behavior of small membrane (1.5 mm diameter) integrated to the microwave resonator sensor in response to flow-rate versus time. (**c)** The variation of resonant amplitude according to different flow-rates in large membrane-integrated microwave resonator (3 mm diameter). (**d)** The variation of the resonant amplitude with respect to different flow-rates in small membrane-integrated microwave resonator (1.5 mm diameter).

The performance of the flow sensor is examined from several aspects, such as sensing range, accuracy, response to flow fluctuation, leaking, reproducibility and long-term detection within the incubator, applicable for further integration into microfluidic-based bioreactors. Applying a pressure to the membrane results in its downward deformation. The release of pressure over the membrane leads to an upward movement to its isovolumetric relaxation, which is demonstrated by the electrical signal of the sensor (Fig. 4). Two circular-shape membranes with the thickness of 100 µm and different diameter sizes of 3 mm and 1.5 mm are tested in flow range of 0–250 µl/min. The size of the membrane can be, however, customized based on the flow-rate range to achieve the highest precision and accuracy. As shown in Fig. 4a, the measured resonant amplitude is scattered and not reliable and repeatable for the large membrane (3 mm diameter) in the flow range below 12 µl/min whereas very repeatable and stable results are observed for flow rates higher than 12 µl/min. The transient measurements are repeated for 5 times and results with associated error bars are presented in Fig. 4c. The instability at low flow rates may be sourced from the high aspect ratio of the membrane (diameter: thickness is 60:1) as this instability is not detected for the smaller membrane (1.5 mm diameter). The small membrane demonstrates reliable performance with clearly distinguishable results at flow rates below 50 µl/min (Fig. 4 b,d). The microwave senor demonstrates a real-time and linear response with both membranes at the desired flow ranges.

**Figure 5:**
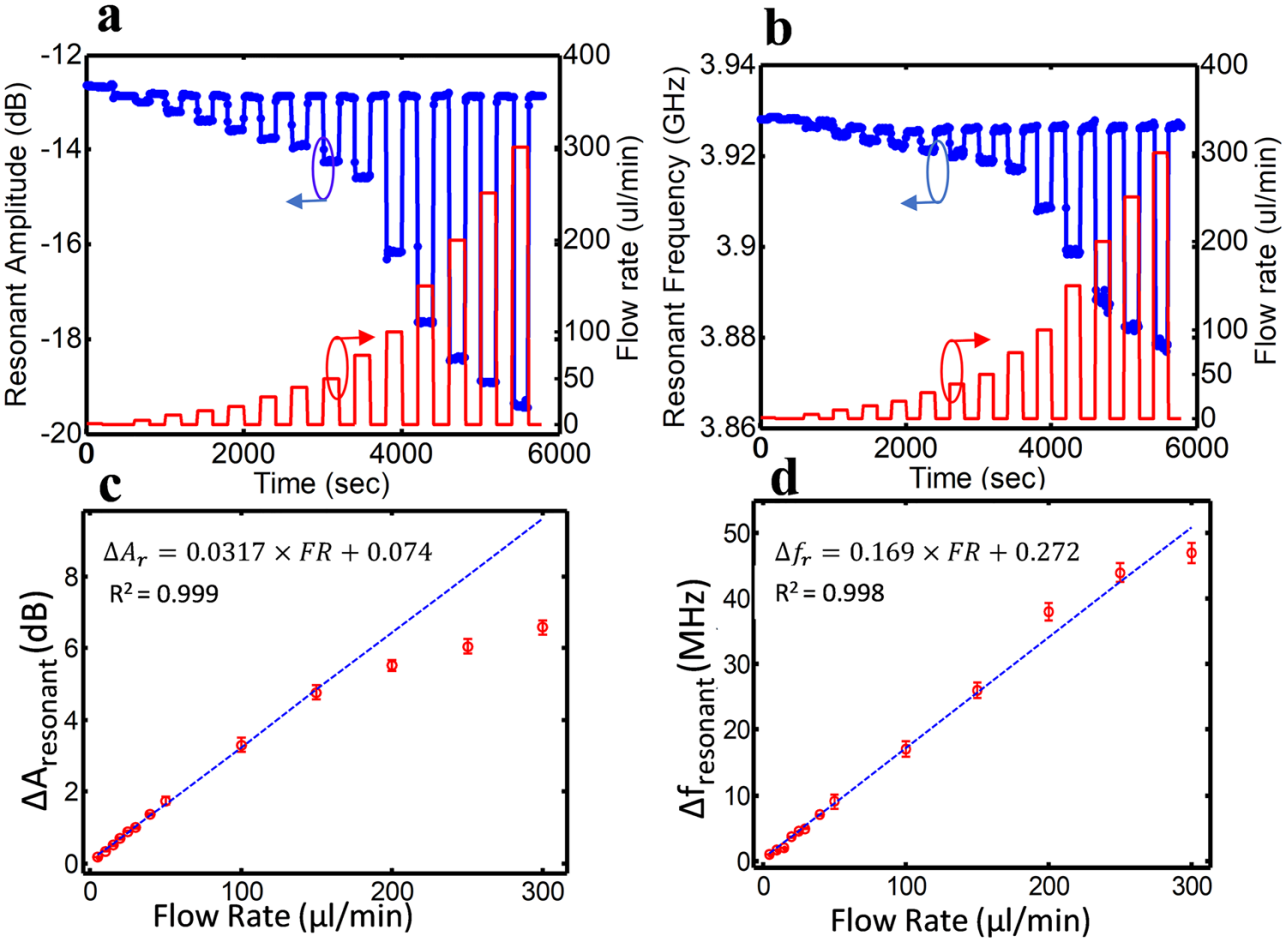
The response of the flow sensor with large membrane (3 mm diameter) to different flow rates following reset condition. (**a)** The transient response of resonant amplitude variation with respect to the flow rate change. (**b)** The transient response of resonant frequency variation with respect to the flow rate change. (**c)** The resonant amplitude against flow rate associated with a fitting curve. (**d)** The resonant frequency against flow rate associated with a fitting curve.

Both resonant amplitude and frequency of the sensor demonstrate variations during the flow against the zero-flow condition. As shown in Fig. 5a and b, the results are repeatable, robust and reliable for the membrane with 3 mm diameter. The flow range is set to 1 to 300 µl/min. The settling time constant is extracted for resonant amplitude and resonant frequency response of the sensor using a curve fitting to the first order exponential equation for each flow rate. During the relaxation period (flow is zero), the time constant is 3+/− 0.2 min. The time constant of the sensor response for the duration when the flow is set to a constant value is flow dependent which is an increasing function of flow rates.

Under the flow experiment, the sensor can detect a maximum flow of 300 µl/min and a minimum flow rate of 0.5 µl/min. The sensitivity of the sensor is defined as the frequency shift over flow variation and determined to be 169 kHz/(µl/min). This sensor demonstrated the ability to detect abrupt flow changes and monitor the flow inside the fluidic network. In the case of priority to the actuation time, the sensor’s respond time could be further improved by modifying the physical properties of the membrane. The 100 times reproducibility testing of the sensor under the flow rate of 5 µl/min in a 20 s/20 s (on/off) manner showed that this flow sensor could accurately and reliably measure the flow rate with the variation of less than 5%. The deformation of the membrane did not generate any bubble during the long-term performance of the sensor which demonstrates its non-disruptive performance to the normal flow of fluids within the channel. Also the membrane in its free standing condition (in absence of the resonator) withstands the deformation of about 1.2 mm under the flow pressure before any occurrence of membrane breakage or leakage at the inlet. This demonstrates that the membrane thickness of 100 µm is a reliable thickness for the effective performance of sensor within the flow rate of 1–300 µl/min.

The experimental results show that the flow sensor with the large membrane (3 mm diameter) functions linearly within the flow rate of 1–150 µl/min, while the linear range response of the small membrane is within the 1–100 µl/min. The nonlinear response of the PDMS material under large deformation and nonuniform change of permittivity above the sensing site may contribute in nonlinear response of the resonator signal for the flow rates of above 150 µl/min. Moreover, the pick and place testing is also performed to assess the sensitive sensitivity to the alignment of microfluidic over that of resonator. The results showed that the pick and place testing has the error below 2% as long as the width of microchannel is less than the gap between the two electrodes.

It is noteworthy to mention that the delay on the response might be due to the fluidic damping factors of the fluidic network from the syringe site to the local sensing point as well as the damping properties of the detection system including the membrane and the electronic system. While the membrane deformation in this work is used for flow sensing, with some modification on the design and incorporation of several circular membranes along the channel, this membrane-based flow sensor can be used for non-contact and non-intrusive measurement of pressure and viscosity of fluids within microchannels. The dielectric constant of the media resulted from the iron concentration, polarization charge and double layer thickness may affect the measured microwave profile and parameters of the flow sensor. Incorporation of two resonators in series or parallel for differential measurements can compensate the dielectric constant of the media, as demonstrated somewhere else^46,47^. The first resonator can measure the liquid permittivity in no bulging site of the channel while the second resonator can measure the microwave parameters right over the bulging membrane. The difference between the measured signals of these two resonators is related only to the bulging effect of the membrane and can measure the flow rate independent from the dielectric constant of the media.

### Long-term performance of the flow sensor in cell culture incubator

**Figure 6:**
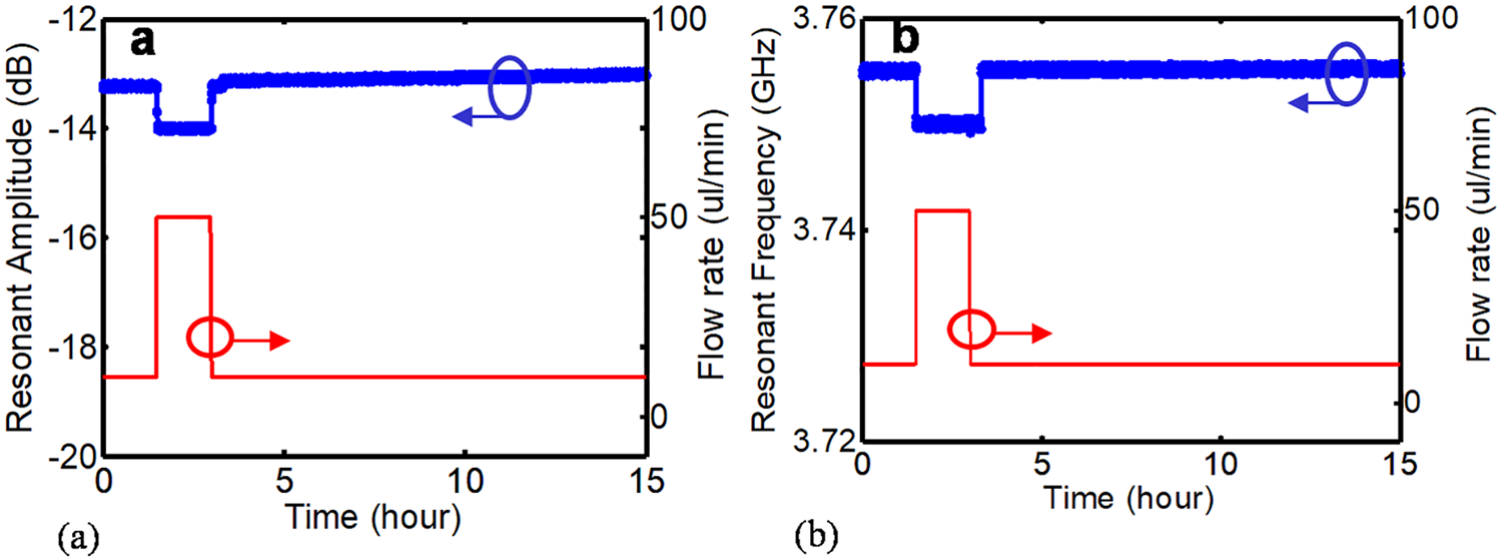
The response of the sensor to two different flow rates of 10 and 50 µl/min in the incubator (37 °C, 5% CO_2_, 100% RH). (**a)** transient response of the resonant amplitude variation with respect to flow rate change. (**b)** transient response of the resonant frequency variation with respect to flow rate change.

The flow sensor is placed inside the cell culture incubator (ThermoFisher) to examine its stability for long-term monitoring the flow rate in microbioreactors in 37°C, 5% CO_2_ environment and 100% relative humidity, and the flow rate is monitored for 15 hrs continuously. The results show that the signal is stable with 2% error. The small signal drift at a few minutes of the detection might be due to the effect of humidity fluctuation on the sensor performance. The initial flow rate is set to 10 µl/min for 100 min and then changes to 50 µl/min for 100 min. To demonstrate the repeatability of the measured results, the flow is set back to 10 µl/min for the rest of experiment (~11 hours). The transient response of the microwave sensor for two flow rates is presented in Fig. 6.

While the flow sensor performance is validated for the flow rates of 1–300 µl/min, the flow sensor can be further modified by changing the dimensions of the membrane or coating stiffer materials like poly(methyl methacrylate) (PMMA) over the thin PDMS membrane to enable detection of much higher flow rates for high pressure liquid and gas detection. The thin layer made of PDMS material in the present study can be fabricated from other biocompatible polymers. However, changing any of these parameters may lead to a different operating flow ranges, and sensor resolution and sensitivity. Also incorporation of several of these membranes at different positions of microfluidic network or high-throughput fluid system enables time to time detection of flow rate and possibly the pressure at any desired channel network using the pick and place method. When designing the microsystem, users can decide the position on which the detection zone is placed to measure the localized flow by leaving the membrane along the desired channel. Also the detection range and sensitivity of the flow sensor developed in this work meet the requirement of OOCs but further improvement can be implemented to enhance the sensitivity and possibly reach the high-resolution sensing within the range of tens of nl/min achieved by SiN or SU8 cantilevers^26^.

## Conclusion

A highly sensitive, noncontact and non-intrusive flow sensor based on integrated microwave- microfluidic technologies is presented. The deformable membrane is fabricated in PDMS and is simply designed as part of the microfluidic network design. The integration of thin film membrane enables monitoring of fluid behaviour. The membrane is designed such that different bulging occurs depending on the flow rate. Consequently, this behaviour is monitored using planar microwave ring resonator. The influence of membrane thickness and diameter on the device performance is characterized. The flow sensor has a linear response in the range of 0–150 µl/min for the optimal sensor performance. The highest sensitivity is detected to be 0.5 µl/min for the membrane with the diameter of 3 mm and the thickness of 100 µm. Further optimization on the membrane diameter and thickness can enhance the sensitivity and detection limit. To the best of our knowledge, this is the first microfluidic-microwave flow sensor that uses the thin layer interfacing for high-performance flow sensors enabling the flow detection without any need to a discontinuity phase like particle or droplet in the flow. Compared to other on-chip flow sensors, this novel flow sensor is non-contact with no interference with the main flow through the microchannels, easy to fabricate, compatible with the multilayer soft lithography fabrication process of OOCs, without requiring much space. It also has the capability of integrability to high-throughput systems to measure the flow rate at several different points of interest with no extra complexity to the chip design or incorporation of bulky optical systems. The membrane remains in deformed state under the flow and does not block the flow pathway, therefore opposed to microcantilever sensors, it introduces minimal fluid noise to the circulatory network. As the primary application, the flow sensor developed in this work is highly well-suited to be integrated with micro-bioreactors and OOCs for long-term detection of flow rate in real-time. This sensor, however, could be useful for a variety of other applications including flow cytometry, cell sorting, nanoparticle synthesis and droplet control within microfluidics.

## Materials and Methods

### Structure and sensing principles

The flow sensor demonstrated in this work consists of a circular membrane that is integrated with a microchannel and placed on top of the sensitive region of the stand-alone microwave resonator (Fig. 1). The flow of the liquid over the PDMS membrane deforms the membrane and alters the effective permittivity of the medium above the sensor. A microstrip ring resonator structure is chosen for the microwave sensor due to its planar configuration and producing a single sensitive spot at its slit. The resonator operates in its half wavelength resonant mode and is coupled to input/output port capacitively. The effective permittivity variation in microfluidic channel is then traced as the frequency shift of the microwave resonator. The PDMS material is chosen for the microfluidic system due to its exclusive features related to biomedical applications including biocompatibility, gas permeability, deformability and chemically inert function. Also PDMS can easily attach to glass and other PDMS layers to make multilayer complex microfluidic devices. It is also a user-friendly material due to is adaptability for creating any type of geometries and thicknesses using replica molding and soft lithography technologies. PDMS is also a low-loss microwave transparent. The device is fabricated with a hybrid low-cost technique which combines the PDMS soft lithography and the printed circuit board (PCB) fabrication processes. The permanent deflection of the membrane deflection under the flow condition in combination with the low stiffness of the PDMS membrane result in a high-performance flow sensor with low power consumption and capability of noncontact detection of the flow rate. No extra on-chip integration of optical or electronic components is required which simplifies the miniaturization, integration and handling.

### Microfluidic chip

The microfluidic chip is fabricated by plasma bonding of the two PDMS layers. The fluidic layers are fabricated by molding the PDMS material (10: 1 weight ratio of prepolymer: curing agent) on SU8 mold using the established protocols^24^. The thick PDMS layer that contains the microchannel design is cast on the mold and baked in an oven for 3 hrs at 80 °C. After curing, the PDMS replica is peeled off from the SU8 mold. The thin PDMS layer that contains the thin circular membrane design is fabricated by spinning a PDMS precursor (10: 1) on a silanized glass slide and cured for 3 hrs at 80 °C. The spinning rotation speed is adjusted to achieve desired thickness of the coated PDMS layer. The inlet and outlet of the microchannel on the thick PDMS layer are punched (ID 1.5 mm) to generate holes for connecting the chip to metal connectors and tubing seals. The two PDMS layers are then aligned irreversibly and bonded with plasma treatment (Plasma Etch PE25) for 45 s at the power setting of 15 W. The assembly is then heated in the oven for 1 hr at 80 °C to strengthen bonding.

The width of the target microchannel for which the flow rate is measured can be independent from the diameter of the circular membrane flow sensor. This enables a high flexibility to integrate the sensor along with a wide range of channel sizes and opens up an avenue for measuring potentially the flow rate at several measurement points in a microfluidic network, applicable to estimate the pressure in the channels. For our experiments, the size of microchannel is fixed to 500 µm × 40 µm × 2 mm for width × height × length. Two different circular membranes with diameters of 1.5 mm and 3 mm are tested. Various thicknesses of PDMS ranging from 10–200 µm are examined but the membrane thickness of 100 µm is determined to be optimal as it is thin enough for high sensitive resonator function and thick enough to withstand the flow pressure generated for the desired range of flow rate. The microchannel is connected to a fluidic inlet linked to the syringe pump while the outlet is connected to the atmospheric pressure. The thin PDMS layer seals the microchannel and acts as an insulator layer between the electronic layer and the fluid to avoid the galvanic contact. The insulator layer also prevents issues associated with double-layer capacitances while preventing the degradation of electrodes and offering benefits for the measurement repeatability and sensor lifetime^48^.

### Simulation of membrane bulging versus flow rate

To determine the effect of fluid–structure interaction on the deformation of circular PDMS membrane in a laminar Newtonian regime, three-dimensional, incompressible Navier-Stokes and continuity simulations are implemented using Comsol Multiphysics as follows:^49^

1 $$\begin{array}{c}\rho \frac{\partial {\bf{u}}}{\partial t}+\rho ({\bf{u}}.\nabla ){\bf{u}}=-\nabla .[p{\boldsymbol{I}}+\eta (\nabla {\bf{u}}+{(\nabla {\bf{u}})}^{{\rm{T}}})]\\ -\nabla .u=0\end{array}$$

Where ρ, η, u, and p are the fluid density, dynamic viscosity, velocity vector field and pressure, respectively. Generally, the structural deformation and deflection of the circular membrane resulting from the moving fluid can be calculated by the displacement-force relationship of an elastic membrane shown in equation (2)^49^.

2 $$F\tau =-n.(-pI+\eta (\nabla u+(\nabla u{)}^{\tau }))$$

where Fτ is fluid loading that consists of pressure and viscous forces and n is normal vector of the boundary. The first term on the right side of equation (2) is the pressure gradient extracted from the fluid simulation results. The second term is the viscous component of the force that depends on the dynamic viscosity and velocity of the fluid. However, given the large deformation of the PDMS membrane under the flow rate of 1–300 µl/min and viscoelastic properties of PDMS material for large deformations, the deflection of the membrane follows the large displacement equation experimentally validated for PDMS material (equation 3)^50,51^.

3 $$w=\,0.474\,\ast \,{[(1-{\rm{\upsilon }})\frac{Pr}{Eh}]}^{\frac{1}{3}}$$

where w is the maximum deflection of the membrane, r is the membrane radius, E and υ are the Young’s modulus and the Poisson’s ratio of the membrane material, respectively, and h is the thickness of the PDMS membrane. The PDMS thin circular membrane is considered isotropic with estimated E and υ values of ∼800 kPa and 0.45^24,48,49^. The velocity on the walls is zero in a laminar flow regime due to the dominant viscous force. The flow rate has a maximum value in the middle of the microchannel and is zero at the walls including the region next to the circular membrane since it is located at the bottom wall of the microchannel. The viscosity of ethanol, water and culture medium is 0.001 Pa.s, 8.9 × 10^−4^ Pa.s, and 0.001 Pa.s, respectively. The viscosity of culture medium remains constant in ambient and culture incubator temperature.

### Microwave flow sensor fabrication

The microwave sensor structure is an open-ended half wavelength ring resonator, fabricated on a high-performance microwave substrate from Rogers (RT/duroid 5880). The substrate has a thickness of 0.79 mm and electrical permittivity and loss factor of 2.2 and 0.0009, respectively. The microwave substrate has copper layers on the top and bottom surfaces as conductive layers with thickness of 35 µm. To transfer the resonator pattern onto the substrate, conventional low-cost printing circuit board technique using chemical etchant is used in room temperature. The implemented resonator has a microstrip structure with two input microstrip signal lines which are coupled electrically to the resonator loop. The microstrip structure has the width of 1.5 mm, the resonant loop of 29 mm and with the coupling gap of 0.3 mm between the signal line and the loop. The fabrication error is less than 5% for minimum features of 0.3 mm. The microwave resonator operates at the resonant frequency of 4 GHz with quality factor of 200 where no PDMS layer is in its near vicinity.

### Simulating the change of electric field of the resonator in response to flow change

To characterize interaction of the electric field of the resonator with the flow inside the microfluidic channel and the bulging of the membrane, a three-dimensional (3D) model is implemented in high frequency structural simulation (HFSS©) software (Fig. 3a). The cross section of the implemented sensor in HFSS is shown in Fig. 3b. The deflecting membrane is placed in the most sensitive region of the resonator, where the electric field is intense and has its maximum value. The electric field in front of the sensor and in a plane vertically aligned with the sensor surface is presented considering no liquid (ε_r_ = 1) within the microfluidic channel. The microfluidic channel layer is considered 400 µm above the sensor surface, where the electric field can interfere with the membrane deflection. The simulation parameters in HFSS software are set as: the maximum number of passes for adaptive solution equal to 30; maximum Delta S of 0.001; and a fast sweep type with the frequency span of 3–4 GHz with 2001 number of points. The simulation is performed in a vacuum box with the boundary condition of radiation for its walls. 50 Ω lumped excitation ports are defined for two signal lines.

### Flow testing

To evaluate the sensing dynamic range and the accuracy of the measurement, a step flow profile is applied with the flow rate of 10–50 µl/min and 200–400 s of holding time (Fig. 4). To test the response of the sensor to flow fluctuations, flow pulses are induced in an increasing, decreasing or on/off manners using a syringe pump. The electrical signal for on and off states are recorded continuously to investigate the response time of the sensor to flow changes. The microwave frequency response is recorded for different flow rates following a zero-pressure (pump off). The data is automatically collected every 10 seconds using a LabView equipped vector network analyzer form National Instruments (VNA NI PXIe-1075). Although the response time of our flow sensor is much smaller than the 10 second collection period we were limited by the data acquisition time required for the VNA and LabVIEW for high precision measurements. All experiments are carried out at room temperature unless otherwise mentioned. To assess the performance of the flow sensor for biological applications, the Dulbecco’s modified eagle medium (DMEM) is flowed into the microchannel over the membrane, and the coupled microfluidic-microwave system is placed inside the cell culture incubator with 37 °C and 100% relative humidity (RH). The flow rate is altered within the corresponding flow range of 1–20 µl/min as used in majority of microbioreactors and OOCs. The liquids are injected into the flow sensor by syringe pump (Harvard PHD 2000).
